# Considering Genomic and Immunological Correlates of Protection for a Dengue Intervention

**DOI:** 10.3390/vaccines7040203

**Published:** 2019-12-03

**Authors:** Joshua Blight, Eduardo Alves, Arturo Reyes-Sandoval

**Affiliations:** 1 Department of Life Sciences, Imperial College London, Sir Alexander Fleming Building, Exhibition Road, South Kensington, London SW7 2AZ, UK; joshua.blight09@imperial.ac.uk (J.B.); eduardo.alves@imperial.ac.uk (E.A.); 2 The Jenner Institute, Nuffield Department of Medicine, University of Oxford, The Henry Wellcome Building for Molecular Physiology, Roosevelt Drive, Oxford OX3 7BN, UK

**Keywords:** dengue, DENV, genomic variation, genomic diversity, T-cell responses, antibody responses, vaccines

## Abstract

Over three billion are at risk of dengue infection with more than 100 million a year presenting with symptoms that can lead to deadly haemorrhagic disease. There are however no treatments available and the only licensed vaccine shows limited efficacy and is able to enhance the disease in some cases. These failures have mainly been due to the complex pathology and lack of understanding of the correlates of protection for dengue virus (DENV) infection. With increasing data suggesting both a protective and detrimental effect for antibodies and CD8 T-cells whilst having complex environmental dynamics. This review discusses the roles of genomic and immunological aspects of DENV infection, providing both a historical interpretation and fresh discussion on how this information can be used for the next generation of dengue interventions.

## 1. Introduction

Infecting over 390 million people each year [1], the dengue virus (DENV) is found worldwide covering over 100 countries (WHO), with either epidemic or endemic occurrence [1]. Its transmission is dependent on the key mosquito vectors, *Aedes aegypti* and *Aedes albopictus* [2,3]. The DENV itself is a positive sense RNA flavivirus which exists as four circulating serotypes [2]. The effects of DENV infection are subclinical in approximately 75% of cases. However, the remainder (over 96 million per year [1]) experience a spectrum typified initially by acute dengue fever (DF) that after defervescence can develop into severe haemorrhagic dengue fever (DHF), characterised by dangerous plasma leakage and hypovolaemic shock which can progress to organ failure and vascular haemorrhage [4,5,6,7,8]. Unfortunately, besides mitigation of symptoms there is no effective treatment for the severe disease [4] and consequently is a major cause of paediatric death and debilitation in many countries (WHO). With cases increasing yearly and steady geographical expansion there is urgent need for effective long-term prophylaxis [1].

Current interventions rely mostly on generic vector-based control [5,6], therefore there is a need for effective interventions specifically targeting the virus. Whilst there are many antiviral therapeutics in development [7,8,9,10,11] they are less attractive as an intervention as viral loads are already declining in symptomatic patients [12,13]. The most desirable intervention is an effective vaccine against the virus. However, due to the complex patho- and immuno-biology of the disease this has proven extremely difficult [14,15]. For example, in natural infections pre-existing immunity to a serotype enhances the infection of subsequent serotypes [4]. As a consequence of this the current and only licensed dengue vaccine was found to enhance the disease in infants [16,17,18,19,20]. It is therefore critical that we work towards a better understanding of the correlates of protection that a next generation dengue intervention must elicit and the dynamics of dengue infection they must target.

## 2. Viral Lifecycle

The DENV depends on both a human and mosquito host to complete its lifecycle, where it exists primarily in urban environments as either endemic or epidemic cycles [21]. The primary urban vector, *Aedes aegypti* is found across the globe in tropical and subtropical regions [2]. The virus also exists in sylvatic cycles in forested areas and has been known to infect humans on rare occasions [22]. Initially an *Aedes* mosquito will become infected by taking a bloodmeal from a febrile human host, following which the virus replicates over a period of 4–7 days [2] before it is found in the salivary gland in high numbers [23]. Following a bite from an infectious mosquito [15] the virus is then released into the human host via the insect’s saliva, which it injects while taking a bloodmeal [24]. The virus has also been shown to transmit maternally in mosquitoes to the female eggs, however its role within urban environments is unknown [25].

**Figure 1:**
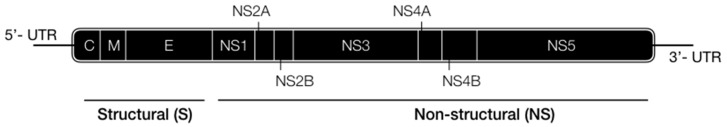
Dengue virus genome schematic.

The virus itself is a 50 nm virion constructed from Capsid (C), preMembrane/Membrane (prM/M), and Envelope (E) protein in a lipid envelope [15]. Inside is a 10.7 kb positive sense capped RNA (+RNA) genome which also encodes seven non-structural proteins (NS1, NS2A, NS2B, NS3, NS4A, NS4B, and NS5; Figure 1) [15].

During human infection DENV has an assorted cell tropism, with a particular preference for dendritic cells, macrophages, and monocytes [15]. Binding and entry of these cells is mediated by the viral E protein [26] and although many host binding proteins have been suggested (heparan sulfate, DC-SIGN, mannose receptor, TIM/TAM receptors, laminin, HSP90/70) the receptor mediating DENV entry remains elusive [15]. The virus enters host cells by clathrin-mediated endocytosis, although there may be alternative entry methods in some cell types [27]. Acidification of the endosomes and the E protein are critical to fusion with the host membrane and release of the viral genome into the cytoplasm. The E protein is a three-domain protein (EDI, EDII, EDIII) and acidification causes it to trimerise to reveal a fusion loop within EDII which mediates this fusion [26,28,29,30]. The EDIII domain distinguishes the four serotypes [14].

Subsequently, the viral RNA is translated by the host machinery as a single polyprotein anchored in the endoplasmic reticulum (ER) membrane [26,31] and the polyprotein is cleaved into its constituent proteins mostly by itself using the NS2B/NS3 protein on the cytoplasmic side, but also by host proteases in the ER luminal side [32,33,34]. NS2B is critical to NS3′s serine protease function [35] and recognition [36]. The RNA genome contains both 3′- and 5′- UTR hairpin loops which modulate viral replication [37]. Processed viral proteins with some host factors form a complex that mediates the replication of the viral genome via a negative-strand intermediate. Most importantly the NS5 RNA-dependent RNA polymerase (RdRp) synthesises the negative-strand RNA which it uses to produce the positive sense RNA with a double stranded intermediate [38,39]. NS3 and NS5 mediate the 5′ capping of the nascent +RNA [39]. NS3 also functions during replication via its helicase activity [40].

The capped RNA is then packaged with the capsid protein and localises with E and prM heterodimers which bud from the ER [31] as immature virions made from 180 prM/E dimers in 60 trimeric complexes [41]. prM acts to prevent the fusion loop from being revealed while in the host [14], however as virions traverse the Golgi prM is cleaved into the pr fragment and M by host furin followed by a realignment of the E proteins into anti-parallel dimers [42]. Following budding from host cells the pr fragment is released, mediated by the change in pH forming mature virions [43]. Furin cleavage is however inefficient, leading to a mixture of prM containing virions [43]. The roles of the remaining non-structural proteins are less well characterised. NS4A is believed to be important in formation of replicative complexes [44], structures where viral RNA replication occurs [45]. NS1 functions in RNA replication and virion production [46]. NS2A and NS4B are also important in replication [47].

## 3. Immune Correlates of Protection

Understanding the correlates of protection for complex pathogens is critical to successful vaccine design in the modern era. They help us to engineer and tailor vaccines to specifically elicit these protective aspects of the immune system in a memory response. Consequently, a rudimental understanding appears only successful for some cases (e.g., smallpox [48]) and may even lead to disease enhancement with life-threatening consequences [49,50]. Unfortunately, dengue falls within the latter group [14,15], and a recent phase III dengue vaccine trial showed increased disease severity in young children [19]. This unfortunate side-effect is however not unexpected. The development of severe dengue following acute fever, epitomised by increased vascular permeability [15,51], almost exclusively occurs during a secondary infection with a different serotype to the primary infection [52,53,54]. Furthermore, infection with each serotype elicits long-term protection to the respective serotype, but only short-term (~2 months–3 years) protection to other serotypes [55,56,57,58,59].

It was therefore hypothesised that infection with one serotype enhanced the infection of a subsequent heterotypic serotype [14,54,56,57]. In support of this, increased viremia was shown to correlate with severe disease [60,61]. In 1988, Srisakul Kliks *et al*. [62] provided direct evidence that maternal dengue antibodies both provided immunity in infants from dengue infection and that their decline correlated with severe dengue symptoms. The lack of T-cells in this situation suggested antibodies as the main mediator of protection and disease severity. Early in vitro work however showed that antibodies could also enhance infection via Fc receptors (FcR) [63].

Since then, considerable work has expanded knowledge on this phenomenon referred to as antibody-dependent enhancement (ADE); described as *cross-reactive IgG antibodies from a previous infection with poor neutralizing capability which bind the heterologous serotype and enhances its uptake* via *FcR’s into host cells* [51,64]. This is likely a mediator of increased viremia mediated through increased host cell infection [64].

Most work has however been inferred from *in vitro* or animal models [63,65,66,67,68,69]. Increased binding and uptake has been shown in P338D1 cells [70] and THP-1 cells [71]. Recent in vitro work suggests that in some FcR-containing cell types the increase in infected cells and viremia in ADE may in fact be mediated by enhanced fusion within endosomes and not increased host cell binding and uptake [70]. Work has also shown that FcR binding in ADE may modulate the antiviral response. For example, THP-1 monocyte cells infected via ADE showed that FcR signalling reduced type I IFN and pro-inflammatory cytokine production, Toll-like receptor (TLR) expression, and nitric oxide radicals, whilst increasing IL-10 expression. This consequently led to enhanced viral production and viral load [71,72,73]. It should be noted however, that IL-10 induction has not been found in macrophages [74]. Considerable work shows or suggests that the high viremia is associated with vascular leakage [72,75,76,77], offering a possible correlate to the severe disease. An *in vivo* mouse model with maternal antibodies, found increased TNFα and linked this to vascular leakage [78]. In humans with severe dengue TNFα levels have been found to be elevated [79,80].

An antibody that causes ADE can be defined as one which binds viral particles but does not prevent viral entry into host cells [51,65]. This is dependent not only on whether it binds to neutralising epitopes, but also circulating levels, its affinity, and how accessible the epitope is [14,64,81,82,83,84]. As such a neutralising antibody could theoretically become disease enhancing in low levels [14,64,81,82,83,84]. A key factor is cross-reactivity (heterotypic) with the secondary infecting serotype, but heterotypic antibodies can be neutralising [85] and heterotypic neutralising antibodies have been associated with reduced symptoms [86].

Severe disease determinants mediated by ADE are therefore multi-factorial and present a complex dynamic process. These include the natural decline of heterotypic neutralising antibodies but not homotypic neutralising antibodies over time [64]. Additionally, the presence of anti-prM antibodies, which may enable non-infectious immature DENV particles with uncleaved M protein (prM) to infect host cells [65,87,88]. DENV particles are also extremely dynamic, undergoing a process known as viral ‘breathing’ which can also affect the ability of neutralising antibodies to bind [4,15].

During a primary infection the majority of antibodies are cross-reactive and non-neutralising (not protective) [66,89], most of these antibodies are anti-E antibodies against the conserved EDII fusion loop [14,89]. During secondary infection, however, anti-fusion loop antibodies are strongly neutralising [85]. Many of the best neutralising anti-E antibodies have been shown to bind complex epitopes dependent on the quaternary protein structure [90,91,92,93,94]. Included within this are antibodies that interact with the fusion loop [90,91,92,93,94].

High levels of poor-neutralising prM antibodies are also found during secondary infection with *in vitro* ADE activity [65,89]. Additionally, antibodies against NS1 are found during infection [65,66,95] and have been shown to enhance NS1′s complement activation activity and cross-react with platelets and endothelial cells. This may be an important contributor to vascular leakage and haemorrhaging [96,97,98,99,100], however, antibodies against NS1 have also been shown to reduce vascular leakage [77]. This may be explained by differences in the amounts or type of antibodies raised against NS1. A few antibodies have also been detected against other proteins, including non-structural [101,102].

In-terms of vaccination this suggests that raising high levels of neutralising antibodies against E would be protective, these could be homotypic or heterotypic. However, a decline in the heterotypic levels could be detrimental. Avoiding prM antibodies may be beneficial to prevent immature virions from becoming infectious. In hindsight it is therefore not surprising that the tetravalent Sanofi CYD-TDV vaccine consisting E and prM caused increased virulence [19]. This could have been mediated by prM antibodies acting on immature virions or the tetravalent nature possibly encouraging cross-reactive antibodies. Alternatively, competition between each virus type may cause certain vaccine serotypes to replicate more skewing the antibody response, which has been seen in mosquitoes and humans [103,104]. It may also explain the differing levels of serotype protection seen [19,105]. However, in a Phase II trial for CYD-TDV, neutralising antibodies against all four serotypes were detected but there was no DENV2 protection [20]. Another consideration is that the titer of the antibody responses against each protein is not uniform (Table 1). Understanding the subtleties of the humoral response elicited by DENV is therefore not only important to avoid detrimental enhancement effects but also to identify potential DENV protein regions with the ability to generate autoreactive antibodies. This should therefore be strongly considered during vaccine design.

Another explanation for the limited vaccine efficacy could be a lack of appreciation for CD8 T-cell responses. As most dominant CD4 epitopes have been found in the, C.; E, and NS3 proteins, whereas most CD8 epitopes have been found in NS3, NS4B, and NS5 [106,107,108,109,110], which are missing from the CYD-TDV vaccine [19]. There are, however, only a few MHCII epitopes currently identified [105,110] therefore more data is needed to confirm a CD4 bias towards, C.; E, and NS3. A summary of the type of responses detected against each viral protein and their humoral intensity is illustrated in Table 1.

Until recently few MHCI epitopes were also known, however the seminal work of Daniela Weiskopf *et al*. [106] increased the number of CD8 epitopes by over 83%, and the Immune Epitope Database and Analysis Resource (IEDB) [123] now has over MHCI 350 human epitopes. This, until recent scarcity of epitopes may partly explain the recent paradigm shift in CD8 correlates, driven partly by the Weiskopf group [105].

Traditionally CD8 T-cells are associated with protection from viral infections via a combination of antiviral activities, including cytotoxicity and the release of pro-inflammatory and anti-viral cytokines, which can be enhanced by CD4 T-cells [105,124,125,126]. However, in dengue, CD8 T-cells have historically been associated with severe disease and capillary leakage, and many refer to Juthathip Mongkolsapaya [127] on this. The only early evidence really excluding a role for CD8s in severe disease was the discovery that maternal antibodies in infants without a pre-existing T-cell response was associated with increased severity [62]. Only recently has there been a considerable increase in evidence for a non-pathogenic or protective role of CD8 T-cells in dengue infection [78,106,128,129,130,131].

The predicted detrimental effects of CD8 T-cells have been referred to as antigenic sin and is defined as *the domination of cross-reactive memory CD8 T-cells from the original serotype during a heterotypic infection which have low avidity* [105]. This proliferation of cross-reactive CD8s instead of naïve CD8s in secondary infection is likely as memory T-cells have a lower activation threshold and are present in higher frequency [132]. The detrimental effects of antigenic sin are theorised to be mediated by the released of vasodilators leading to vascular leakage [133,134]. For example, T-cell secretions such as IFNα and IL-2R have been correlated with disease severity [80], however the concentration of blood products due to plasma leakage may confound such results [69]. Furthermore, although TNFα has been implicated in disease [135], it has also been implicated in disease in primary infection of mice with maternal antibodies [78]. This data suggests that pre-existing cross-reactive CD8s may not be a predisposing factor.

Most of the literature report dengue specific CD8 T-cells during the acute phase [127,128] and thus before the onset of severe disease, providing the premise that they could cause pathogenesis [128]. Commonly groups will, however, refer to the work by Nguyen Thi Phuong Dung *et al*. [69] who only detect dengue specific CD8s after the onset of vascular leakage. Although tempting, this does not necessarily mean that CD8s play no role in pathogenesis as they may be sequestered in tissues or enhance vascular leakage in late stages [69]. Alternatively, these differences may reflect host genetic backgrounds as all use the same NS3 epitope and HLA-A11 MHC [69,127,128]. This does highlight the restraints of using only a restricted number of HLA and epitopes to draw conclusions.

Interestingly, Heather Friberg *et al*. [128] reported that dengue specific CD8 T-cells reach peak frequency slightly earlier in primary than secondary infection. This observed difference may be explained by increased apoptosis of CD8s in secondary infection, which has been reported using an NS3 epitope measuring Ki67 positive cells [127]. In further support of a pathogenic CD8 role, an epitope- and HLA-independent measure for CD8 proliferation (CD96) has shown that during the acute febrile phase there is an increase in total activated CD8 T-cells in patients that developed vascular leakage [136]. Heather Friberg *et al.* [128] however showed no difference in the frequency of dengue specific CD8 or their activation level (CD38) in acute phase for primary or secondary infection and used multiple epitopes and HLAs.

In further support of antigenic sin, evidence exists to suggest that cross-reactive MHC binding does not necessarily elicit an effective CD8 response [132,137]. These cross-reactive CD8s are clearly able to bind epitopes across serotypes and in many cases due to serotype variability (25%–30%) these will have sequence differences (epitope variants) [132]. Considerable work on epitope variants (mostly with the NS3 GTS epitope and its variants) have shown that although stimulation with a heterotypic epitope variant (an epitope variant from another serotype) induces proliferation these T-cells are low avidity [127] and have reduced cytotoxic degranulation with elevated cytokine release [132]. Furthermore, Heather Friberg *et al.* [137] extensively characterised the activation of dengue specific CD8 T-cells and showed that some heterotypic epitope variants showed an atypical mono-functional CD8 response characterised by MIP-1b release, whereas other heterotypic variants of the same epitope and the homotypic variant were associated with a polyfunctional response (MIP-1b, degranulation, TNFα, IFNγ) [137]. A poly-functional response is associated with protection in other viruses [138,139]. Consequently, this suggested that the heterotypic serotype (and therefore heterotypic epitope variant) that a person gets challenged with could actually affect the type of cross-reactive CD8 response they get, i.e., the order of serotype infection [137]. In support of this, greater numbers of dengue specific CD8s producing TNFα, IFNγ, and IL-2 prior to heterotypic infection was associated with an asymptomatic infection [130].

Additionally, Heather Friberg *et al*. [137] suggested that as the release of the CD8 mediators was sequential starting with MIP-1b, that the mono-functional response maybe due to inherent features of the epitope variant sequence effecting the length of TCR interaction. A process referred to by the Weiskopf group [105] as altered peptide ligands (APL) [140]. It has been suggested that cross-reactive CD8s may preferentially bind to heterotypic epitope variants [127], however this is not the case and instead epitope preference is due to inherent properties of the peptides affecting HLA avidity [128].

Finally, the retrospective study of Daniela Weiskopf *et al.* [106] which identified considerable numbers of new CD8 epitopes, showed that during primary infection most CD8 targeted serotype-specific epitopes, however this changed to mostly targeting cross-serotype conserved epitopes in secondary infection with a different serotype. Therefore, there is some skewing of CD8 responses dependent on what epitopes are conserved between the two serotypes you have been infected with. However, these conserved epitopes were of high avidity and produced a poly-functional response (INFγ, IL2, TNFα). Although, they did conduct a meta-analysis which linked HLA as a risk factor in disease severity which was associated with low CD8 response magnitude, showing that a low magnitude response was associated with a mono-functional response.

The combination of supporting evidence for both a protective and detrimental role for CD8 suggests that CD8 may play a multifaceted role in disease, which may depend on CD8 epitope characteristics in the context of epitopes variants and level of antigen presentation enhancement by ADE. This may have detrimental implications for the other two most progressed vaccines from NIH and Takeda which contain dengue proteins targeted by both antibodies and CD8 T-cells [105,141]. Furthermore, the tetravalent nature of these vaccines [141] may promote cross-reactive CD8 against epitope variants with unpredictable responses. The evidence therefore suggests that a vaccine based on cross-serotype conserved CD8 epitopes may more predictably raise protective poly-function CD8 responses.

An alternative/additional explanation for the considerable contradiction and confusion with the literature regarding CD8 function during dengue infection is likely due to the different methods, HLA’s and epitopes used, the quick dynamics of immune cells [128,137], lack of standardised assays [14], and the problem with obtaining human samples especially at different time points during infection (acute or severe). A final consideration is the limited availably of animal models to study infection which can fully simulate a human infection [78].

## 4. Viral Dynamics

Although early serological work identified four distinct circulating dengue viruses (DENV1, 2, 3 and 4), more recent genetic analysis has identified distinct genotypes within each serotype which consist of further clades [142]. These are commonly geographically separated [142], however some ‘cosmopolitan’ lineages are found in multiple continents [142,143,144] and in some cases more than one lineage is present in the same region [145].

Dengue dynamics in endemic regions are complex, characterised by continual lineage turnover and changes in prevalence [146,147]. These lineage turnover dynamics have however been classified on a genetic level as following two non-mutually exclusive patterns, (i) an incremental spectrum of dead-end variants within lineages and (ii) dramatic replacements of lineages [144,147]. Recent phylogenies based on E genome sequences have identified five genotypes in DENV1 and 2, and four genotypes in DENV3 and 4 [142,148].

**Figure 2:**
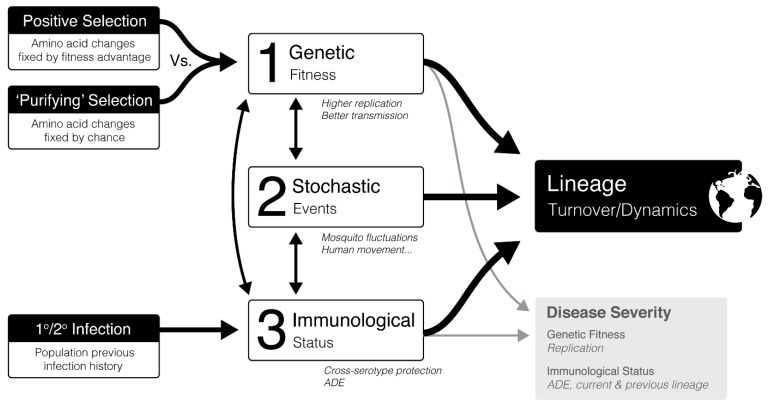
Dengue dynamics and its implications for vaccine design. Lineage turnover and dynamics depend on three key factors; genetic fitness, population immunological status, and stochastic events. (1) Genetic fitness linked to increased transmission and out competition of other lineages has been associated with positive selection of non-synonymous mutations [142,144,146] and purifying/negative selection by introduction of mutations fixed by chance that have fitness advantages in an immunological context [144]. (2) Stochastic events such as mosquito population fluctuations, human movement, and seasonality may influence lineage dynamics [142,153]. (3) The immunological status of the population in context of previous dengue infection likely effects lineage dynamics by balancing between ADE-mediated infection enhancement and short-term cross-serotype protection [153,155,156,157]. Both viral replication rates and previous serotype/lineage infections have been associated with disease severity [146]. ADE; antibody-dependent enhancement.

DENV lineages have been associated with varying disease severity [149] and transmission rates [147,150], therefore an understanding of these dynamics would greatly aid future intervention methods including vaccine design. However, in the field there is considerable controversy regarding the factors modulating lineage dynamics, with many papers supporting alternative hypothesises [142,144,146,147,150,151,152,153]. Although, most agree that as yet we do not have a firm enough grasp to predict these dynamics. Within the field, three different factors have each been hypothesised to control dengue dynamics; (i) genetic fitness, (ii) population immunological status, and (iii) stochastic events, each with supporting evidence from the field [142,144,146,147,150,151,152,153]. This controversy is likely a consequence of the multifactorial nature of dengue infection dynamics. Coincidently, recent work [142,144,146,154] is starting to appreciate that all three factors likely contribute in a complex cross-talk. Using the current sphere of knowledge, a hypothetical diagrammatic representation of this complex dynamic process has been created (Figure 2), which is discussed in detail below.

**(i) Genetic fitness** is one of the most controversial areas. This is the idea that ‘fitter’ or more ‘virulent’ strains will outcompete and increase in prevalence and replace the weaker lineages (positive selection) [147]. A fitter virus can be seen as one with greater transmission, which is theorised to be related to increase replication in the vector or host and thus more ‘virulent’ [158,159]. Recent work supports this showing that more virulent South East Asian DENV2 strains outcompete less virulent strains in mosquitoes co-infected with both [160].

Importantly, increased DENV viral replication has been associated with more severe disease [60,161]. Evidence for positive selection and its association with virulence has been reported. For example, a replacing clade in Thailand in the 1990s was found to have higher viral titers within the mosquito hemocoel, suggesting increase transmission [147]. Furthermore, the replacing DENV2 lineage in Vietnam during the late 1990s was associated with increase viral titers in humans which was linked to increased transmission [150]. In Nicaragua a replacing DENV2 clade had nine amino acid substitutions not found in the old clade, two of which were found in the NS5 RdRp region which could affect its RdRp activity (R401K and T290I) [146]. Positive selection of non-synonymous amino acid substitutions, assessed by maximum-likelihood, has also been associated with replacement clades. For example, minor positive selection has been identified for NS2A [144,162,163] E protein [143,154] and NS1 [152]. Mutations in E, for example, may affect cell tropism or cell fusion [154].

Positive selection of mutations is not surprising since RNA viruses lack a proofreading RdRp and are estimated to introduce one mutation per replication [142,150]. However, paradoxically and controversially, most the DENV genome has been shown to be under purifying selection, shown by low non-synonymous (dN/dS) substitution rates [151,164]. It is hypothesised that having to replicate alternately in two hosts puts significant genetic restraints on the virus, hence purifying selection. In support of this a group showed that the virus, cycling in 10 alternating passages between insect and mammalian cells caused an increase in fitness in their respective cells [163]. This was not seen with one cycle alternating culture, indicating that using two hosts restricts fitness to balance effective replication in both hosts [142]. This suggests that mutations are deleterious to survival and therefore ‘purified’ [152]. Consequently, this provides support for use of a conservation-based approach to design a vaccine.

Groups that support purifying selection (amino acid changes fixed by chance) suggested that clade replacements are instead due to stochastic bottlenecks [145,165,166,167,168]. **(ii) Stochastic events** likely playing a contributing role in lineage dynamics include human population movements, mosquito population dynamics, and other environmental factors [144,169]. Indicating that controlling these may have implications for vaccine efficacy by effecting the pool of genetic variants (i.e., lineages) that hosts need to be protected against.

However, it has been suggested that some amino acid substitutions fixed by chance during purifying selection, i.e., not positively selected, could actually be associated with a fitness advantage when an **(iii) immunological selection pressure** is present within the population [144]. Although serotype protection is serotype-specific [4], cross-protection between serotypes has been reported for a short period after a DENV infection [4,146,153]. In support of this hypothesis a study in Thailand in the late 1990s found that a new DENV1 clade replacement was associated with an overall decline in DENV1 prevalence, but increase in overall DENV4 prevalence, suggesting that the surviving DENV1 clade may have had initial less cross-protective immunity with DENV4 than the preceding DENV1 clade [144]. In further support of this, they found that amino acids changes between the two DENV1 clades were not under positive selection [144]. Furthermore, it has been suggested that the replacement of DENV1 and 2 in Iquitos with DENV3 was due to low population immune protection against DENV3, which lead to increased DENV3 infections, which in turn lead to short-term cross-protection against DENV1 and 2 [170].

Conversely, an increase in prevalence of lineages has been associated with infection enhancement by ADE in secondary infection using models [171], which coincidently has been linked to increased chances of severe disease [146]. In support of this, OhAinle *et al.* [146] showed that a replacing DENV2 clade in Managua was associated with more severe disease. Interestingly, they found that disease severity risk was associated to which DENV2 clade they became infected with following a primary DENV3 infection [146]. Intriguingly a model which includes cross-protection suggested that it is cross-protection which causes the more unpredictable dramatic lineages changes [153]. Models supporting both these scenarios have predicted that ADE enhancement and serotype cross-protection are large factors controlling dengue lineage dynamics (including replacement) and prevalence [153,155,156,157,172]. This considerable evidence for positive selection above, and this, suggest that both positive and purifying selection likely play a key role in dengue dynamics.

## 5. Current Vaccine Strategies

Early attempts at vaccinating against dengue have failed due to the risk of disease enhancement, where pre-existing antibodies from an earlier serotype cross-react with poor neutralizing ability against different serotypes in a subsequent infection [4]. The reason for this is the inclusion of B-cell epitopes and the use of tetravalent approaches. The recently and only approved tetravalent live attenuated chimeric dengue vaccine (CYD-TDV) by Sanofi Pasteur [173], also known as Dengvaxia^®^ is believed to suffer from the enhancement phenomenon as it showed an increase in the risk of severe dengue in infants below five years [16,17,18,19,20] and increase enhanced disease in vaccinated seronegative population regardless of age [174]. It is based on four chimeric yellow fever viruses with the structural genes replaced by DENV structural genes [18] and therefore aims to elicit neutralizing antibodies. It does, however, exclude NS1 in an attempt to avoid pathogenic effects [175]

The enhancement seen with CYD-TDV is a major concern to the many other tetravalent vaccines also in development, all of which contain regions of the DENV proteome known to elicit robust antibody responses [105,141]. Two of these are currently undergoing phase III trials; the attenuated DENVax (Takeda; http://clinicaltrials.gov/show/NCT02747927) containing four chimeric constructs based on a DENV2 backbone, and the TV003/TV005 (NIAID; http://clinicaltrials.gov/show/NCT02406729) based on four attenuated serotypes [173]. Both contain CD8 inducing non-structural dengue proteins that are absent from Sanofi CYD-TDV, however, they also contain structural proteins raising the concern of eliciting enhancing or autoreactive antibodies. Furthermore, the tetravalent nature of these vaccines raises the question of how you can ensure each construct raises similar level immune responses to prevent enhancing effects against some of the serotypes.

In flavivirus seronegative adults, TV003/TV005 did however, elicit a near-sterilising immunity [171]. Nevertheless, the estimated date for study completion of these second-generation dengue vaccines are not expected until late 2021 and 2025 for TDV and TV003/TV005, respectively therefore the long-term effects are not yet known. A CD8 T-cell only vaccine would avoid antibody mediated disease enhancement, however they are currently none published, even though recent evidence suggests they could be highly protective [106,111,137].

## 6. Conclusions

The clinical outcome of dengue infection (1 or 2°) in individuals depends on a considerable set of factors both (i) dengue specific, such as dengue immunological status (previous infections, order of infections) [105], genetic fitness of the lineages infected [147,149,150], and (ii) patient specific, such as age, HLA status, and unknown genetics [133]. Understanding the factors that determine the clinical outcome of a disease and the correlates of protection are key to the development of effective interventions.

In the context of dengue these factors are complex, and although still not fully understood, mean that interventions must be specifically tailored to not only be effective against all four serotypes but to avoid detrimental enhancement effects in subsequent DENV infections or based on previous infections. Once such example of this is the Sanofi antibody-based vaccine which showed limited efficacy and elicited ADE in some trial participants [19].

Therefore, the key to the future of dengue eradication will be the use of novel designs and approaches with a significant theoretical founding. In this review, we have summarised many of the key correlates that must be considered when designing future dengue interventions, incorporating both immunological and genomic data for a rational design.

**Figure 3:**
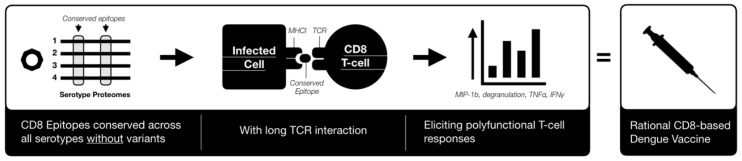
A monovalent CD8 Dengue vaccine. CD8; cluster of differentiation 8.

Based on this there is considerable evidence [105,106,137], although mixed [127,132] which suggests that eliciting a carefully targeted CD8 response, possibly one with MHCI interactions involving long TCR engagement that elicit polyfunctional responses and/or against epitopes conserved across serotypes without variants [106,127,137] could achieve this goal (Figure 3). Key benefits of this approach include the avoidance of antibody responses which may enhance infection or have autoreactivity to host. It also avoids the use of a tetravalent approach which increases the chances of raising unequal immune responses against each serotype due to differences in replication of each virus within a tetravalent vaccine. Although including CD4 epitopes such as ones raising neutralising antibodies against E would be protective, you need to be able to maintain high titers to prevent disease enhancement. Additionally, by using CD8 epitopes conserved across serotypes you ensure all genetic lineages are covered, avoiding the replacement of circulating lineages with more virulent lineages. Whilst avoiding CD4 epitopes also avoids ADE-mediated lineage replacement. Such an example candidate shows how a greater understanding of dengue correlates of protection could help to achieve the ideal cross-serotype prophylactic dengue vaccine and we hope that this review encourages further thoughts on the development of new and distinct dengue interventions.

**Definitions**

- ADE: The process where cross-reactive IgG antibodies from a previous infection with poor neutralising capability bind the heterologous serotype and enhances its uptake via FcRs into host cells.
- ADE Antibody: Antibody that binds viral particles but does not prevent viral entry into host cells.
- Antigenic-Sin: The domination of cross-reactive memory CD8 T-cells from the original serotype during a heterotypic infection which have low avidity.
- Heterotypic: A different DENV serotype.
- Homotypic: The same DENV serotype.
- Cross-reactive: Immune cells which react to multiple serotypes.
- Epitope Variants: Epitopes across serotypes with sequence differences between serotypes.
- Purifying Selection: Amino acid changes fixed by fitness advantage.
- Positive Selection: Amino acid changes fixed by chance.

## Figures and Tables

**Table 1:** Dengue virus genomic regions and their human immunological relevance.

Region	Function	T-cell Epitope [111]	B-cell Epitope [112]
5′ UTR [113,114] 5′ untranslated regions	Assists viral RNA replication by direct binding with NS5 and stabilizes long range RNA–RNA interactions	---	1° Response: none 2° Response: none
C [115] Capsid Protein	Assists nucleic acid rearrangements, crucial for nucleocapsid formation	CD4, CD8	1° Response: none 2° Response: +
M [116] Membrane	Structural role in forming parts of the prM-E complex	---	1° Response: + 2° Response: +++
E Envelope	Key role in interaction of DENV with host cells	CD4	1° Response: +++ 2° Response: +++
NS1 [46] Non-structural 1	Acts on immune evasion by binding into host complement proteins, modulates early events in RNA virus replication by interacting with virus dsRNA and NS4B protein.	---	1° Response: +++ 2° Response: +++
NS2A [47] Non-structural 2A	Antagonises the host immune response, component of the viral replication complex and plays a role in virus assembly	CD4	1° Response: none 2° Response: +
NS2B [117] Non-structural 2B	Regulates viral protease activity (co-factor of the NS3 protease)	CD4	1° Response: none 2° Response: +
NS3 [118] Non-structural 3	Acts as protease, RNA helicase and RTPase/NTPase	CD4, CD8	1° Response: + 2° Response: ++
NS4A [44] Non-structural 4A	Induces host membrane alterations important for virus replication	CD4, CD8	1° Response: none 2° Response: +
NS4B [119] Non-structural 4B	Assists viral RNA replication through direct interaction with NS3	CD4, CD8	1° Response: none 2° Response: +
NS5 [120,121] Non-structural 5	Fundamental role in viral genome amplification, counteracts antiviral response through STAT2 degradation	CD4, CD8	1° Response: none 2° Response: +
3′ UTR [122] 3′ untranslated regions	Assists long range RNA–RNA interaction between the ends of the viral genome	---	1° Response: none 2° Response: none

The major targets for human T-cell and B-cell epitopes with DENV. The main human primary and secondary antibody responses for each structure are marked with crosses: + (low titer), ++ (moderate titer), and +++ (high titer). DENV; dengue virus.
